# Limited geographic variation in the acoustic structure of and responses to adult male alarm barks of African green monkeys

**DOI:** 10.1007/s00265-014-1694-y

**Published:** 2014-03-06

**Authors:** Tabitha Price, Oumar Ndiaye, Kurt Hammerschmidt, Julia Fischer

**Affiliations:** 1 Cognitive Ethology Laboratory, German Primate Center, Göttingen, Germany; 2 Courant Research Centre for the Evolution of Social Behaviour, Georg August University of Göttingen, Göttingen, Germany; 3 Applied Behavioural Ecology and Ecosystem Research Unit, UNISA, Preller St, Pretoria, 0002 South Africa; 4 Direction de Parc National de Niokolo-Koba, Velingara, Senegal

**Keywords:** Alarm call, *Chlorocebus*, Geographic variation, Language evolution, Vervet monkey, Vocal communication

## Abstract

The global diversity of human languages is a remarkable feature of our species, which requires a capacity for rapid vocal learning. Given that primate alarm calling systems have played an important role in the language origin debate, identifying geographic variation in primate alarm calls and understanding the underlying causal mechanisms are important steps to help uncover evolutionary precursors to language. This study investigates geographic variation in the alarm bark of the widely distributed African green monkey (*Chlorocebus*). To quantify geographic variation in spectral and temporal call structure, acoustic analysis was used to compare the adult male barks of green monkeys (*Chlorocebus sabaeus*) and two subspecies of vervet (*Chlorocebus pygerythrus pygerythrus* and *Chlorocebus pygerythrus hilgerti*). Playback experiments were also carried out to test whether adult male vervets would distinguish between the barks of own-group males, unknown conspecific males and green monkey males. Acoustic analysis showed that, whilst similar in overall structure, the barks of green monkeys could be distinguished from vervet barks with a high degree of accuracy; the barks of vervet subspecies could also be discriminated, although to a lesser degree. Males responded most strongly to unknown conspecific males’ barks, and exhibited responses typical of leopard-avoidance and territorial defence. Taken together, these findings indicate that variation in alarm calls can be best explained by phylogenetic distance, and that intra- and inter-species differences are relevant during social interactions. Moreover, barks may function as an alarm and display call, which could explain the observed sexual dimorphism in barks in this genus.

**Electronic supplementary material:**

The online version of this article (doi:10.1007/s00265-014-1694-y) contains supplementary material, which is available to authorized users.

## Introduction

Human speech displays extensive regional differences in the language spoken, dialect and accent, and it has been proposed that the emergence of vocal learning was a critical step during the evolution of the modern language faculty (Janik and Slater 1997; Oller and Griebel 2008). Vocal communication in nonhuman animals also exhibits geographic variation (Weilgart and Whitehead 1997; Slobodchikoff et al. 1998; Bradbury et al. 2001; Davidson and Wilkinson 2002; Smith and Hunter 2005; Delgado 2007), but whilst speech patterns are strongly influenced by learning, the ability to produce novel vocalisations as a result of experience has been identified in only a few other distantly related taxonomic groups (Janik and Slater 1997). In nonhuman primates (hereafter primates), vocal learning is notably scarce and may be limited to the modification of existing vocalisations as a result of social interaction (reviewed in Egnor and Hauser 2004; Hammerschmidt and Fischer 2008). Such vocal modification is often inferred when population differences in vocal structure cannot be explained by genetic or habitat structure (Fischer et al. 1998; Crockford et al. 2004). Studies investigating the causal mechanisms underlying vocal variation in primates are therefore a first step towards a better understanding of the factors that drive acoustic divergence, and ultimately vocal learning.

Between-species variation in primate loud-call structure is generally attributed to genetic differences (Oates and Trocco 1983; Brockelman and Schilling 1984; Méndez-Cárdenas et al. 2008; Wich et al. 2008; Thinh et al. 2011; Meyer et al. 2012), and species-specific differences in such calls can be used as a non-invasive tool for discriminating between cryptic species (Zimmermann et al. 2000). Despite the important role that primate alarm calls have played in the search for the roots of human language, and having been identified as a flexible, context and audience-dependent behaviour requiring sophisticated cognitive processes (Zuberbühler 2007), far less is known about geographic variation in primate alarm calls. In a recent review, Wilkins and colleagues (2013) introduced a framework for the comparative study of acoustic divergence across a broad range of taxa. Within this review, they identified three scenarios under which acoustic characteristics may change in relation to (neutral) genetic distance. Firstly, acoustic and genetic distance may covary as a result of genetic drift; secondly, acoustic characteristics may diverge more rapidly due to sexual selection in association with ecological differentiation or a genetic mutation related to signal production and species recognition, and finally acoustic signals may diverge slowly at first and then rapidly increase in variation if the costs of hybridisation drive reproductive character displacement. Clearly, broad comparative acoustic analyses including phylogenetic reconstruction are needed to identify the evolutionary processes giving rise to observed acoustic variation. Quantifying the geographic variation present in alarm calls within and between closely related primate species, and investigating how vocal variation affects receiver responses will contribute to a clearer understanding of the dynamics of primate vocal evolution.

**Fig. 1:**
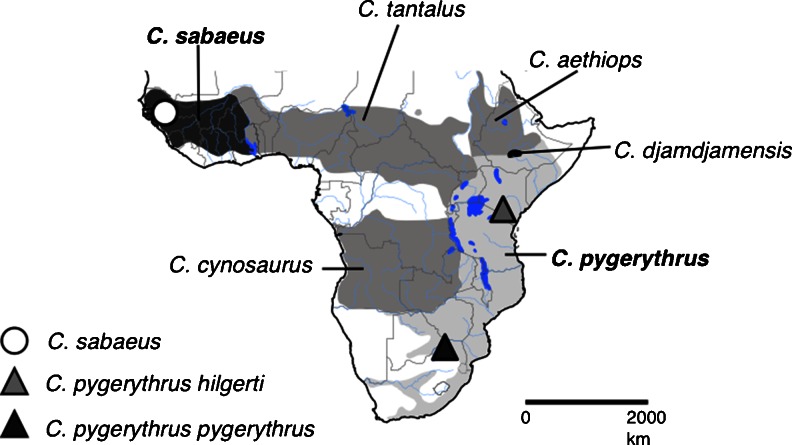
Distribution of African green monkeys (*Chlorocebus*) and sites at which recordings were made for analysis of call structure. Species distributions are *shaded* and modified from Lernould (1988) and Kingdon (1997)

African green monkeys (genus *Chlorocebus*) provide an excellent model to study such processes. This group of monkeys is one of the most widespread African primates, distributed over much of sub-Saharan Africa (Lernould 1988; see Fig. 1). Extensive differences in pelage length and colouration have been recorded across this range (Dandelot 1959; Hill 1966; Napier 1981) indicating the presence of genetic variation across the genus, and these differences have been used to split the genus into four monotypic and two polytypic species (Groves 2001, 2005). Whilst taxonomy within the genus is still disputed (Grubb et al. 2003), recent analyses of mtDNA diversity clearly separate the green monkey (*Chlorocebus sabaeus*) in the West from all other species (Haus et al. 2013) and propose that the initial split within African green monkeys occurred between this Western clade and all other lineages approximately 2.81–2.76 MYA (Wertheim and Worobey 2007). Analyses of mtDNA also support genetic separation within the polytypic vervet (*Chlorocebus pygerythrus*) taxon, between mainland subspecies *Chlorocebus pygerythrus hilgerti* ranging from Ethiopia to northern Tanzania, and *C. pygerythrus pygerythrus* in Southern Africa (Haus et al. 2013). This variation can be attributed to a more recent period of rapid diversifications within the genus, occurring approximately 1.59–1.48 MYA (Wertheim and Worobey 2007).

Despite these morphological and genetic differences, vocal comparisons amongst African green monkey populations were, until now, limited to qualitative descriptions. These observations suggested that vocalisations are spectrally stable among vervet subspecies and between vervet and tantalus (*Chlorocebus tantalus*) populations, but that they may exhibit differences in temporal characteristics (Struhsaker 1970). A quantitative analysis of intra- and inter-species vocal differences and how African green monkeys respond to the vocalisations of other populations was, however, lacking. This is somewhat surprising, as the alarm calls of East African vervets constitute one of the most well-known examples of so-called functionally referential signals (Seyfarth et al. 1980), and studies of geographic variation in this genus may offer insights into the flexibility present in their vocal behaviour, and thus shed light on the mechanisms that give rise to context-specific calling.

In this study, we carried out structural analyses of bark calls produced by adult male South African vervets (*C. pygerythrus pygerythrus*), adult male East African vervets (*C. pygerythrus hilgerti*) and adult male green monkeys (*C. sabaeus*) to investigate vocal variation between and within species, including the variation between individuals within the South African population. Following this, we tested how South African adult male vervets perceive the barks of own-group males and unknown males of the same subspecies, and unknown male green monkeys. The adult males of all three populations have been reported to produce barks in response to terrestrial predators, and playback experiments have shown that East African vervets frequently respond to conspecifics’ alarm barks by climbing a tree, an appropriate leopard-avoidance behaviour (Seyfarth et al. 1980). Similar leopard-avoidance behaviour was observed in green monkeys, with subjects always climbing more than 2 m into a tree in responses to a leopard model (Price and Fischer 2013). Thus, if South African vervets recognise intra- and inter-specific bark calls as indicating the presence of a terrestrial predator, they should respond by climbing to a height of more than 2 m into a tree. However, acoustically similar barks are also given during aggressive encounters (Struhsaker 1967; Galat and Galat-Luong 1976; Cheney and Seyfarth 1992), which frequently entail males chasing after another male (TP personal observation).

The aim of this study was thus to identify the degree of variability present with the structure of bark calls, to investigate how this variability is perceived, and to offer insights into the function of adult male barks. Given the limited evidence for vocal learning in nonhuman primates (Hammerschmidt and Fischer 2008), we predicted to find little variation between and no variation within species. Considering the presumed stabilising selection acting on alarm calls (Struhsaker 1970), and the costs of not responding to a putative alarm call, we expected calls to cause males to climb into trees, and furthermore we expected relatively little variation in listeners’ responses to the playback of calls with different origins.

## Geographic and individual differences in call structure

### Data collection

Adult male bark vocalisations were recorded from green monkeys, East African vervets and South African vervets at three geographically distant study sites within the range of African green monkeys (Fig. 1). All study subjects were habituated to human presence and were recognised individually from natural markings on the face and body. The barks of East African vervets were recorded by Thomas Struhsaker (June 1963–May 1964), and Robert Seyfarth and Dorothy Cheney (1977–1988) as part of their earlier studies on several free-ranging groups within the semi-arid acacia savannah of Amboseli National Park (2°39′49 S; 37°15′16 E) in Kenya, and these calls were subsequently made available for inclusion within this study. Green monkey barks were recorded by TP over two field seasons (January–June 2010 and 2011) from four free-ranging groups and two solitary males within Niokolo-Koba National Park (13°01′34″ N, 13°17′41″ W), an area in southeastern Senegal consisting mainly of Sudano-Guinnean savannah interspersed with woodland and gallery forest (Frederiksen and Lawesson 1992). South African vervet barks were recorded by TP and ON (January–June 2012) from five free-ranging groups located within the Loskop Dam Nature Reserve (25°25′18S; 29°18′29E) in South Africa, which contains a mixture of open grassland, acacia dominated woodland and low mountains with open tree savanna (Filmalter 2010).

In all studies, adult male barks were recorded ad libitum when the context of calling could be confirmed as the presence of a feline terrestrial predator, either by observing the predator or hearing its vocalisations. Whilst the natural occurrence of bark calls was not uncommon, it was often not possible to confirm whether a terrestrial predator was present at these times. Following numerous studies that have successfully used the presentation of predator models to elicit alarm calling (e.g. Coss et al. 2007; Arnold et al. 2008; Wheeler 2010), spontaneous barks from all field sites were supplemented with barks produced in response to leopard models. Altogether, five different leopard models were used. East African vervet calls were recorded at a distance of 0.5 to 7 m using a Nagra III or a Nagra SNN tape recorder at 9.5 cm/s and MKH 804 Sennheiser directional microphones (R. Seyfarth and D. Cheney) or an Uher 4000 Report-S portable tape recorder at 7 ½ ips with a frequency response of about 40–20,000 cps (manufacturer’s specifications), parabolic reflector and a Shure dynamic microphone (T. Struhsaker; no information on distance available for these calls). These calls were later digitised at 22.05 or 44.1 kHz with a 16-bit resolution. South African vervet and green monkey calls were recorded by TP at a distance of 3 to 10 m using a digital Marantz PMD661 solid-state recorder (44.1 kHz sampling rate, 16bits accuracy) connected to a Sennheiser ME66/K6 directional microphone.

### Call selection

Bark vocalisations were frequently produced in long calling bouts. Consecutive bark elements (the basic units represented by a continuous sound) were identified using the pulse-train analysis of Avisoft SASLab Pro (version 5.1.17) and the start and end points of each element were recorded. From these labels, we calculated inter-unit intervals and used a log survivor function (Slater and Lester 1982) to determine a time threshold of 75 ms, below which elements were classified as belonging to the same call. On the basis of this, bark calls can be made up of one or more bark elements, with inter-call intervals exceeding all intra-call intervals and multi-unit barks frequently containing first exhalation (Ex1), within-call exhalation (Ex2) and inhalation (Inhal) call elements (definition of terms modified from Struhsaker 1967). It was not possible to analyse all calls from a calling bout, as males were often too far away from the microphone when they started calling and/or many calls were overlapped by the calls of conspecifics. As such, call samples were taken from as near as possible to the start of each calling bout, but had to be tailored to the number and quality of calls recorded.

To investigate population-level differences in adult male bark vocalisations, we analysed barks from 12 green monkeys, 12 South African vervets and 13 East African vervets. Unless insufficient calls of adequate quality were available, 10 bark calls were selected from the calling bout of each individual, resulting in a total call sample of 352 bark calls (120 green monkey barks, 120 South African vervet barks and 112 East African vervet barks). To investigate individual-level differences, we analysed the bark vocalisations of six adult male South African vervets. We could not extract uninterrupted whole calls from all bouts of each individual, and as such we were not able to test temporal differences at the level of the whole call. To identify differences in element structure, we selected 20 Ex1 bark elements from five calling bouts per male, resulting in a call sample of 100 bark elements per male and a total of 600 bark elements.

### Acoustic analysis

**Fig. 2:**
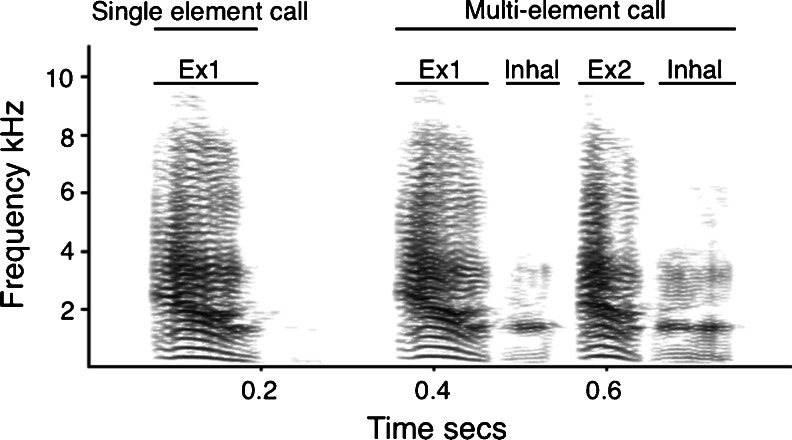
Adult male bark series illustrating a single element and a multi-element call. Labels indicate temporal characteristics and different element types. The spectrogram was created using Avisoft SASLab Pro, with a 512 FFT and a Hamming window

For analysis of population differences, call duration, Ex1 duration and the number of elements within a call were calculated based on the call labels described above (Fig. 2). For analysis of population and individual differences, spectral analysis was carried out on Ex1 call elements only. Call processing prior to spectral analysis was carried out using Avisoft SAS Lab Pro. Calling bouts were first highpass filtered at 80 Hz to remove background noise below the lowest frequency of calls, following which, undisturbed Ex1 bark elements of high signal-to-noise ratio were extracted and padded with silent margins. Next, the frequency and temporal resolution of calls was adjusted to optimise measurement accuracy; for robust measures of energy distribution throughout the call unit, sampling frequency was reduced to 16 kHz, and calls were transformed using a fast Fourier transformation (FFT) size of 1,024 points, Hamming window and 93.75 % overlap. These same settings were used to extract a measure of tonality, but because calls frequently only exhibited tonality at low frequencies and higher frequency noise hindered calculations, calls were first lowpass filtered at 1.2 kHz. For measures relating to fundamental frequency (F0), sampling frequency was reduced to 8 kHz and calls were transformed using an FFT size of 1,024 points, Hamming window and 96.87 % overlap. The resulting frequency-time spectra were analysed with LMA, a custom software sound analysis tool developed by KH, and parameters used for analysis are described in Table 1.

**Table 1:** Description of the acoustic parameters used to describe the temporal and spectral structure of barks

Measurement	Description
Temporal	
Duration (ms)	Duration of call
Ex1 duration (ms)	Duration of single or first call element
Element number	The number of elements per call
Spectral	
F0 (Hz)	Mean fundamental frequency across all time segments
F0 start (Hz)	Fundamental frequency at the start of the call unit
F0 end (Hz)	Fundamental frequency at the end of the call unit
F0 linear trend	Factor of linear trend of fundamental frequency
Tonality (%)	Percentage of tonal time segments for which F0 can be calculated
First_quartile (Hz)	Median first frequency quartile across all time segments
First quartile_1–4 (Hz)	Mean first frequency quartile at 1st, 2nd, 3rd and 4th temporal quartiles
Second_quartile (Hz)	Median second frequency quartile across all time segments
Second quartile_1–4 (Hz)	Mean second frequency quartile at 1st, 2nd, 3rd and 4th temporal quartiles
Third_quartile (Hz)	Median third frequency quartile across all time segments
Third quartile_1–4 (Hz)	Mean third frequency quartile at 1st, 2nd, 3rd and 4th temporal quartiles
Frequency range (Hz)	Mean frequency range
Peak frequency (Hz)	Median peak frequency across all time segments
Peak frequency_1–4 (Hz)	Mean peak frequency at 1st, 2nd, 3rd and 4th temporal quartiles
PF linear trend	Factor of linear trend of peak frequency
PF deviation (Hz)	Mean deviation between peak frequency and linear trend

### Statistical Analysis

To assess population and individual differences in male bark calls, we first applied a stepwise method to identify a subset of optimum variables for each classification. We set population or caller identity as the grouping variable and entered all temporal and spectral parameters into a stepwise variable selection using the stepclass function of the R package “klaR” (Weihs et al. 2005) with leave-one-out cross-validation. To assess the degree to which barks could be correctly assigned and to determine which structural properties contributed most to differentiating between the different populations or callers, we then entered the selected variables into a linear discriminant analysis (LDA) using the lda function of the R package “MASS” (Venables and Ripley 2002) with a jack-knife leave-one-out method. We compared the classification results of the LDA to those of a nested permuted discriminant function analyses (pDFA; Mundry and Sommer 2007) to control for the pseudo-replication introduced by using multiple calls from a single calling bout. All statistical analyses were carried out using R (R Development Core Team 2011), and we ensured that assumptions were met (see S1-S2 in Online Resource 1) before running tests.

### Results

Stepwise variable selection identified a subset of five acoustic parameters that best differentiated between calls from different populations. These parameters were: Ex1 duration, F0start, F0 linear trend, frequency range and PF deviation. On the basis of differences in these parameters, LDA (using a leave-one-out method) correctly classified 82 % of bark units to their population of origin; this result was supported by the pDFA, which also correctly classified 82 % of calls. Calls were most distinct at the species level, with 96 % of calls being assigned to the correct species, compared to 77 % of vervet calls that were assigned to the correct subspecies (Table 2).

**Table 2:** Percentage of calls assigned to each population and descriptive statistics (mean ± SD) of acoustic parameters used for classification

	Call assignment			Acoustic parameters				
	Green monkey	South Vervet	East Vervet	Ex1 duration duration	F0 start	F0Linear trend	Frequency range	PF deviation
Green monkey	94 %	3 %	3 %	262 ± 110	254 ± 40	−0.14 ± 0.1	2197 ± 708	128 ± 68
South Vervet	1 %	78 %	21 %	99 ± 16	282 ± 57	−0.11 ± 0.2	3116 ± 831	101 ± 53
East Vervet	4 %	22 %	74 %	113 ± 19	320 ± 61	−0.10 ± 0.2	3565 ± 645	156 ± 115

**Fig. 3:**
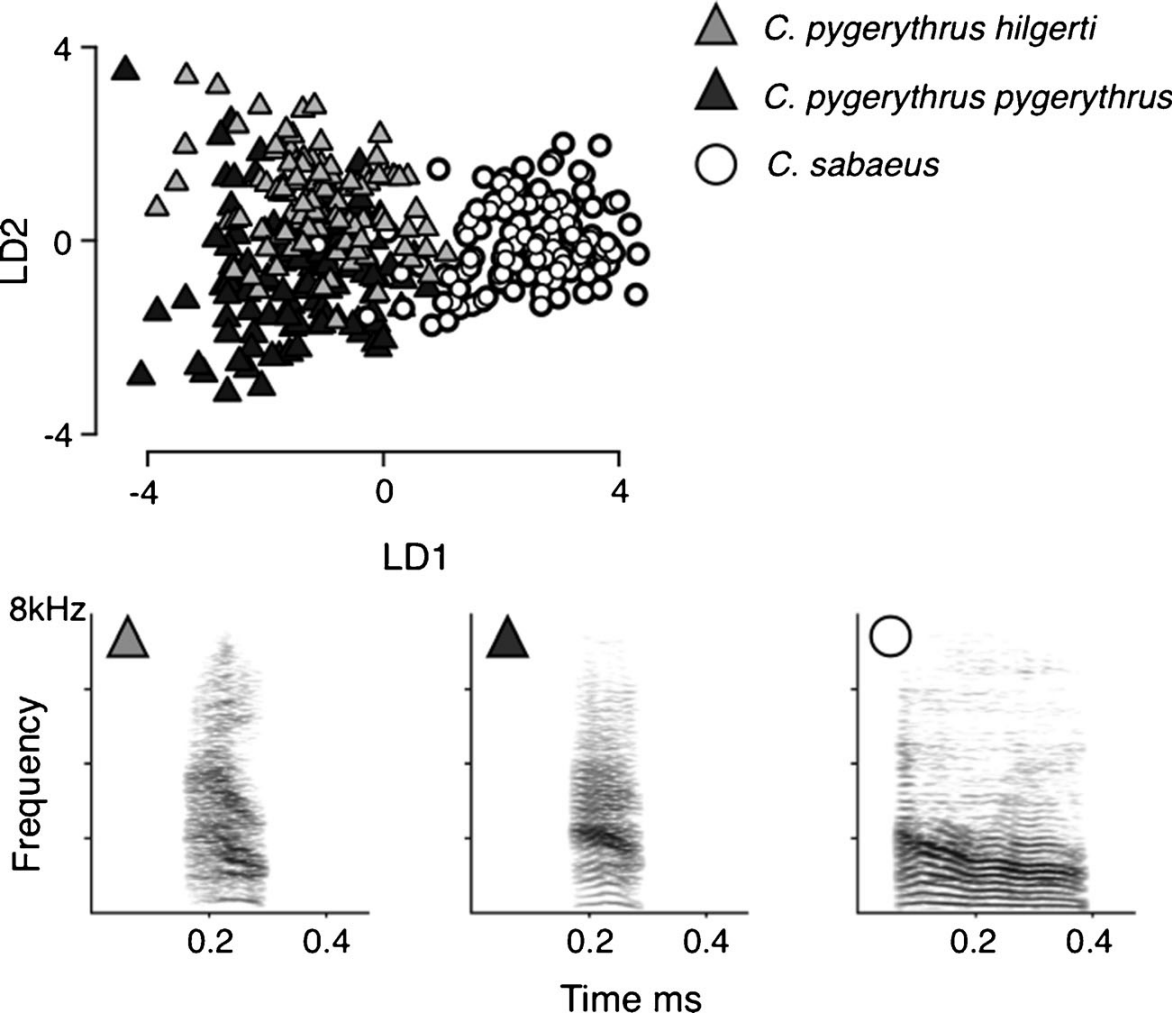
Scatterplot and spectrograms illustrating population differences in the acoustic structure of *C. sabaeus*, *C. pygerythrus hilgerti* and *C. pygerythrus pygerythrus* barks. The scatterplot presents the distribution of the first and second LDA discriminant scores. Spectrograms illustrate a typical call exemplar for each call group, with typical calls defined as those that were most likely to be assigned by LDA to the correct caller/population. Spectrograms were made with a 512 FFT and a Hamming window

The first discriminant function separated green monkey from vervet barks and accounted for 90 % of the total variance explained. Ex1 duration contributed most to this classification, with West African green monkeys typically producing longer barks than vervet monkeys, although the Ex1 duration of green monkey calls was also quite variable. The second discriminant function accounted for 10 % of the total variance explained, and separated South and East African vervet calls. This discriminant function was most dependent on differences in F0 start, Ex1 duration and frequency range (Table 2, Fig. 3).

Stepwise variable selection carried out to look at individual differences in the acoustic structure of Ex1 call elements identified five acoustic parameters to enter into a subsequent LDA. These parameters were F0, first quartile_1, first quartile_2, first quartile_4 and Ex1 duration. Entering these parameters into LDA (using a leave-one-out method) resulted in 70 % correct assignment (range = 59–94 %) of South African vervet barks, very similar to the 71 % correctly assigned with pDFA. The first discriminant function described 74 % of the total variance explained and was most influenced by F0. The second discriminant function described 17 % of the total variance explained and was most influenced by measures of the first quartile energy band.

## Behavioural responses to bark vocalisations

### Experimental protocol

Playback experiments were conducted with South African vervets between January and June 2012 by TP and ON. Study subjects were 11 habituated and individually recognised adult male South African vervets from four free-ranging groups within Loskop Dam Nature Reserve in South Africa. For each experiment, we played back bark calls elicited by a leopard model. These barks originated from own-group adult males (“South-own”), unknown adult males of the same subspecies (“South-unknown”) and adult male green monkeys (“West”). Barks used as stimuli for the West and South-own playback conditions were selected from recordings of green monkeys and South African vervets that were used in the structural analysis of bark calls. Barks used as stimuli for the South-unknown playback condition were recorded by Daniel van der Post from free-ranging groups in the Okavango Delta (18°25′42 S; 21°53′39 E) and Limpopo (22°54′25 S; 28°2′28 E) both in Botwana, and from Lajuma Research Centre (23°2′21S; 29°26′58E) in South Africa. Playback stimuli were made up of 6–12 barks units with a high signal-to-noise ratio that were produced as single and double unit exhalations sometimes interspersed with inhalation units. All bark units were taken from a single calling bout, with inter-call intervals, call compositions and maximum amplitude held constant between conditions. Reflecting temporal differences identified in the above section of this study, however, mean duration of call elements was longer in West playback stimuli than in South-unknown and South-own stimuli.

Barks were played back to male vervets using a within-subjects design such that, with one exception, each of the eleven subjects experienced one playback of each condition. This exception was due to a male migration which left one study subject as the sole male of his group before a final experiment could be carried out. We therefore conducted a total of 32 playback experiments (11 West, 11 South-unknown and 10 South-own). The order of playback trials was balanced across conditions, and to avoid habituation effects, playback experiments were carried out on one group with a minimum separation of 6 days. To avoid pseudo-replication, a different playback sequence was used for each playback experiment. As much as possible, each call sequence was produced by a different individual, and no individual contributed calls to more than two playback stimuli.

Playbacks were initiated when the study subject was sitting resting on the ground or low down (<2 m) in a tree, and when the caller (in south-own condition) or another adult male (south-unknown and west conditions) was out of sight. This was to control for the subject responding to observed male presence rather than to identity cues present in the broadcast call. Playbacks were not carried out within 60 min of the natural production of bark calls or any other alarm calling within earshot of the experimenter. In addition, since we were interested in the propensity of the subject to climb up into a tree, it was necessary that at the time of the experiment, there was a tree of >2 m in close vicinity to the subject. Prior to an experiment, the loudspeaker was hung using a net bag behind a natural obstacle at 1–2 m from the ground and 42–59 m from the study subject. Playback stimuli were broadcast using a Marantz PMD-661 solid-state recorder connected to a loudspeaker (David Active, VISONIK, Berlin), with maximum amplitude set within the range of natural calling behaviour (66–79 dB at 10 m from source, measured using a Voltcraft 322 sound level meter). Experiments were discarded if the subject moved prior to stimulus presentation (*n* = 1) if there were technical problems with the equipment (*n* = 2), if the individual was lost before the end of the experiment (*n* = 1), or if the subject’s behaviour was altered by the presence of human food (*n* = 1).

### Behavioural analysis

Following each playback, a single subject was filmed for at least 30 s using a Sony Handycam (DCR-HC90E). At the end of these 30 s, we recorded whether the subject had climbed more than 2 m up into a tree, and, using a tape measure, the maximum horizontal distance travelled. Thirty seconds was selected because it was the maximum time period that all subjects were visible on film, importantly all subjects that responded by climbing up into a tree did so well within this time frame (maximum 8 s after the playback stimulus). To assess behavioural effects over a longer time period, subjects were followed for 30 min (from the point at which the playback was broadcast), and at each 5-min interval, we recorded their height (as being more or less than 2 m from the ground), and their position using a handheld GPS (Garmin GPSMAP 60CSx). We additionally took GPS points of the position of the loudspeaker, the subject’s initial position at time of playback, and of the subject’s position 3 min after the playback experiment.

Post-experiment, we used GPS points to calculate the distance that a subject moved relative to the loudspeaker within the first 3 min (distance from speaker at 3 min, minus distance from speaker at start) to assess whether the subject would approach the speaker on hearing the calls, a behaviour typical of territorial defence; 3 min was selected as the time frame to capture the subject’s early response based on behavioural observations of time until male left the initial position. GPS points were also used to calculate the amount of time an individual spent at more than 2 m from the ground within 30 min of the playback (using a total of 6 height measures taken at each 5-min interval for each experiment), a typical behaviour when a leopard is present. We selected a time frame of 30 min because responses to leopard presence are often sustained over a longer time frame, and this was the maximum amount of time possible to follow all subjects. After each experiment, we also filmed the starting position of the subject with ON pointing to show the direction of the playback speaker. Videos were subsequently imported into Adobe Premiere Pro CS4 with a time resolution of 25 frames/s, and frame-by-frame analysis of videos was used to score the duration of the subject’s first orientation towards the speaker, defined as an orientation within 45° of the direction indicated in the film footage. Because video encoding is susceptible to observer-bias, 50 % of the videos were reanalysed by a second condition-naive observer. Inter-observer reliability demonstrated moderate agreement (intra-class correlation coefficient 0.7). A description of behavioural measures is given in Table 3.

**Table 3:** Description of the behavioural measures used to describe subjects’ responses to playback experiments

Behavioural measure	Description
Strength of response:	
First orientation (s)	Duration of first orientation towards loud speaker
Initial displacement (m)	Maximum distance travelled within 30 s of experiment
Leopard-appropriate response	
Arboreal escape	Does subject climb to >2 m within 30 s of experiment
Time arboreal	Is subject >2 m high within the 30 min following experiment
Male-Male competitive response	
Initial approach (m)	Distance approached towards loudspeaker 3 min post-experiment
Minimum approach (m)	Minimum distance to loudspeaker within 30 min of experiment

### Statistical analysis

To test whether bark origin would have an effect on the strength of response, we used a general linear mixed model (GLMM) with Gaussian error structure to assess differences in the duration of subjects’ first orientation towards the playback speaker, and a GLMM with Poisson error structure to assess differences in initial displacement. We ran GLMMs with binomial error structure to test the effect of bark origin on leopard-typical response behaviours, more specifically looking at differences in whether the male climbed immediately up into a tree (arboreal escape) and whether the male spent more time up in a tree over the next 30 min (time arboreal). Lastly, to test whether bark origin would have an effect on the male-male aggressive behaviours, we used GLMM with Gaussian error structure to analyse differences in the distance approached towards the speaker after 3 min (initial approach) and the minimum distance to loudspeaker over the next 30 min (minimum approach).

For all GLMMs, playback condition (west, south-own, south-unknown) and sequence order were entered as fixed effects and subject was entered as a random effect; to test for differences in subjects’ height over the 30-min period, experiment was added as an additional random effect to account for interval data being included as separate data points. Maximum likelihood was used to achieve more reliable *P* values for models run using a Gaussian error structure. Likelihood ratio tests were used to compare full models with null models (comprising only of sequence order and the random effect), and Markov Chain Monte Carlo sampling using the functions pvals.fnc and aovlmer.fnc of the R package languageR (Baayen 2011) was applied to calculate *P* values for the different levels for behaviours that differed between playback conditions. All models were fitted in R using the function lmer of the R package lme4. For details of test assumptions, see S3 in Online Resource 1.

### Results

The majority of playbacks (30/32) elicited an orienting response, with the male looking immediately in the direction of the speaker, whilst fewer playbacks (13/32) elicited immediate (within 30 s) displacement, for further details of the occurrence of these behaviours, see Table 4. With regards to the strength of response, there was a significant effect of playback condition on the males’ first orientation towards the speaker. In response to barks of South-unknown origin, males’ first orientation was significantly longer than first orientation towards barks of own-group males (likelihood ratio test: −4.7 ± 1.5, t = −3.2, P_MCMC_ < 0.01), and first orientation towards green monkey barks (likelihood ratio test: −4.7 ± 1.4, t = −3.3, P_MCMC_ < 0.01). There was no significant effect of playback condition on males’ initial displacement, or on leopard-typical response behaviours. Looking at male-male aggressive behaviours, playback condition did not affect whether subjects immediately approached the loudspeaker (initial approach), but there was a significant effect on minimum approach, with males approaching closer in response to south-unknown stimuli than to south-own (likelihood ratio test: 21.4 ± 5.8, t = 3.7, *P*_MCMC_ < 0.01), or West African calls (likelihood ratio test: 13.4 ± 5.6, t = 2.4, *P*_MCMC_ < 0.05). The occurrence or descriptive statistics for these behavioural measures, and the results from GLMMs are presented in Table 5.

**Table 4:** Occurrence of orientation, initial displacement and initial approach behaviours

Behaviour	Own	Unknown	Green
Orientation towards speaker	8/10	11/11	11/11
Initial displacement	5/10	4/11	4/11
Initial approach	1/10	4/11	3/11

**Table 5:** Description of response behaviours measured as mean ± SD or occurrence, and results from general linear mixed models for the six behavioural variables measured during playback experiments

Behaviour	Own	Unknown	Green	*χ* ^*2*^	df	*P*
First orientation (s)	2.1 ± 2.0	5.3 ± 2.9	1.8 ± 1.6	11.92	2	<0.01
Initial displacement (m)	1.0 ± 1.6	3.8 ± 7.1	1.9 ± 4.2	2.19	2	0.33
Arboreal escape	3/10	3/11	2/11	0.38	2	0.83
Time points arboreal	35/60	43/66	28/66	3.03	2	0.22
Initial approach (m)*	5.1 ± 10.4	-2.5 ± 15.0	0.5 ± 11.9	2.14	2	0.34
Minimum approach (m)	53 ± 18	29 ± 15	42 ± 22	11.15	2	<0.01

*Negative values represent an approach towards the speaker

## Discussion

### Population differences in call structure

Despite their overall acoustic similarity, the bark calls of West African green monkeys, East African and South African vervets could be distinguished on the basis of spectral and temporal differences in call structure. Of the three populations, correct classification at the species level was high (96 % of calls). The calls of vervet subspecies were also distinguishable, although to a lesser degree, being correctly classified in 77 % of cases, indicating that call differences were larger between than within species. This finding is not suggestive of vocal learning, which leads to rapid changes in vocal structure independent of genetic differences between populations. In addition, vocal differences are unlikely to have arisen as a result of adaptation to the local acoustic niche, because all three populations inhabit mixed savannah woodland, with larger variation in habitat type within than between sites. Instead, results are in accordance with phylogenetic data which identifies green monkeys as especially distinct from other *Chlorocebus* taxa (Haus et al. 2013) with an estimated divergence time of 2.76–2.81 MYA, compared to 1.59–1.48 MYA divergence of vervet subspecies (Wertheim and Worobey 2007). Thus this study indicates that, unlike geographic variation in patterns of human speech, acoustic divergence in the structure of the African green monkey bark is likely the result of phylogenetic distance rather than learning processes. A similar correspondence between acoustic divergence and phylogenetic distance was found for the long calls of leaf monkeys (*Presbytis* spp., Meyer et al. 2012) as well as the song of crested gibbons (*Nomascus* spp.,Thinh et al. 2011).

In contrast, the alarm calls of western grey and eastern rufous mouse lemurs (*Microcebus murinus* and *M. rufus*), despite diverging 6.7–7.3 MYA (Weisrock et al. 2012), do not differ (Zimmermann et al. 2000). One explanation for these differences could be that African green monkey barks, similar to the loud calls of leaf monkeys and crested gibbons, also play a role outside of alarm contexts (Struhsaker 1967), and they may therefore be less subjected to stabilising selection than the more stimulus-specific mouse lemur alarms.

With regards to how African green monkey barks differ, this study identified the duration of Ex1 bark elements as the most influential parameter distinguishing between green monkey and vervet barks, with green monkeys producing longer, but also lower frequency, call elements than vervets. East African vervets on the other hand produced barks with a lower fundamental frequency and a larger frequency range than their South African sister taxon. The production of calls with lower frequency bands and lower F0 could well be the result of a larger body size (and corresponding larger vocal anatomy), a correlation found to hold across a wide range of primate species (Hauser 1993). Support for this explanation comes from a comparative study of cranial measurements across the genus which identifies green monkey samples as being larger than those of vervets (Elton et al. 2010). Interestingly, patterns of skull size within the genus map to clinal variation in rainfall, and it has been hypothesised that larger body size is influenced by differences in habitat productivity (Cardini et al. 2007). Size differences between South and East African vervets are, however, unclear (Pasternak et al. 2013). The prolonged bark produced by male green monkeys could also be the result of a larger body size in relation to lung capacity (Fitch and Hauser 2002), but it seems unlikely that this could account for such large differences in call duration. If, as suggested above, male barks function as a sexual display as well as an alarm call, the longer bark duration could be a sexually selected trait.

### Behavioural responses to bark vocalisations

The duration of the subject’s first orientation towards the speaker was significantly longer in response to unknown conspecific calls than to known conspecific calls, strongly suggesting that adult male vervets differentiate between the calls of known and unknown males and are more attentive to the presence of “strangers.” That males differentiate between these call categories is supported by the earlier finding of this study that male barks exhibit individual differences in call structure. This result does not, however, require that males recognise group members individually (i.e. “true” individual recognition; Tibbetts and Dale 2007). That males are more attentive to unknown calls is not surprising, as vervet monkeys live in relatively stable multi-male multi-female groups (Fedigan and Fedigan 1988) and actively defend their territory against intruding males (Struhsaker 1967). Additionally, subjects’ longer orientation to unknown conspecific barks than to unknown inter-specific barks suggests that inter-specific differences in this call are relevant in inter-group relationships. We predicted that subjects should respond to all barks with the leopard-typical response of climbing up into a tree. We found that subjects climbed into a tree on hearing the playback stimulus in 8/32 experiments only, with no effect of playback condition. Thus, our results show that conspecific and inter-specific barks elicit leopard-typical escape responses, demonstrating some degree of the predicted constraint that receivers should be under to respond appropriately to alarm calls. Nevertheless, the finding that arboreal escape responses were elicited in only 25 % of trials suggests that, at least for adult males, contextual cues such as conspecifics’ behaviour at the time of the experiment or the subject’s prior experience may also play a role in the attribution of meaning to perceived barks. Interestingly, on hearing the playback stimulus, males also approached the playback speaker in 25 % of trials, suggesting that barks may also function as an intra-sexual display and elicit territorial defence behaviours. In addition, males were more likely to move nearer towards the playback speaker in the 30 min following experiments after hearing the calls of an unknown conspecific than after hearing the calls of a known conspecific or hetero-specific male, corroborating the assumption that males are able to differentiate between the calls of own-group and unknown males, and conspecific and hetero-specific males, and are more attentive to the calls of unknown males of the same species.

## Conclusion

Subtle variation in the structure of the African green monkey bark can be used to differentiate between species and to a lesser extent subspecies. This strengthens the general consensus that phylogenetic differences account for a large degree of the vocal variability found within and between primate species, not only in loud calls but also in calls produced in alarm contexts. Future studies comparing geographic variation in call types that are either subject to sexual selection or not, will provide insights into the evolutionary dynamics of acoustic divergence in primates. From the perspective of bark perception, this study suggests that adult males differentiate between the barks of own-group and unknown males, and respond more strongly to unknown conspecific than unknown heterospecific barks. Thus within and between-species variation in bark structure appears to be important in regulating inter-group relationships, or relationships among males. That males respond to barks both by climbing into the trees and by approaching the speaker suggests that adult male barks may have a dual function; a terrestrial alarm call and a male display. More generally, this study supports the hypothesis that, in contrast to human speech, flexibility in primate vocalisations is shaped by call function.

## Electronic supplementary material

Below is the link to the electronic supplementary material.

Online Resource 1 (DOCX 22 kb)
