# Supplementary material for: Limited geographic variation in the acoustic structure of and responses to adult male alarm barks of African green monkeys
Source: Behav Ecol Sociobiol. 2014 Mar 6;68(5):815–25. doi: 10.1007/s00265-014-1694-y (PMC3986895; doi:10.1007/s00265-014-1694-y)
Supplement: Supplementary file 1 — (DOCX 22 kb) [file 265_2014_1694_MOESM1_ESM.docx]

**Behavioral Ecology and Sociobiology**

**Limited geographic variation in the acoustic structure of and responses to adult male alarm barks of African green monkeys**

Tabitha Price^1,2,3*^, Oumar Ndiaye^4^, Kurt Hammerschmidt^,1^, Julia Fischer^1,2^

^1^ Cognitive Ethology Lab, German Primate Center, Germany

^2^ Courant Research Centre for the Evolution of Social Behaviour, Georg August University of Göttingen, Germany

^3^ Applied Behavioural Ecology and Ecosystem Research Unit, UNISA, South Africa

^4^Direction de Parc National de Niokolo-Koba, Tambacounda, Senegal

*Contact: [tprice@dpz.eu](mailto:tprice@dpz.eu)

**S1. Geographic differences in call structure**

Before running LDA to assess how call structure varied between populations we visually inspected histograms of the 31 acoustic parameters to check that there were no outliers and that their univariate distributions were approximately symmetrical. Predictor variables that were not symmetrically distributed were transformed. When taking the log or square-root of the parameter did not achieve symmetrical distribution, we used the Box-Cox function (R package "mass", Venables and Ripley 2002) to find the optimum transformation based on maximum-likelihood methods. After transformation all acoustic parameters were standardised using a Z-transformation in order to make them more comparable in terms of their units of measure and variance. See Table S1 for which transformation was applied to each parameter.

| **Parameter** | **Transformation** |
| --- | --- |
| Duration | Log |
| Element number | None |
| Ex1 duration | ^ -0.8 |
| F0 | None |
| F0start | None |
| F0end | ^ -0.1 |
| F0 linear trend | None |
| Tonality | +3 then log |
| First quartile | None |
| First quartile_1 | None |
| First quartile _2 | None |
| First quartile _3 | None |
| First quartile _4 | None |
| Second quartile | None |
| Second quartile _1 | None |
| Second quartile _2 | None |
| Second quartile _3 | None |
| Second quartile _4 | Log |
| Third quartile | None |
| Third quartile_1 | None |
| Third quartile _2 | None |
| Third quartile _3 | None |
| Third quartile _4 | None |
| Frequency range | None |
| Peak frequency | None |
| Peak frequency_1 | None |
| Peak frequency_2 | None |
| Peak frequency_3 | None |
| Peak frequency_4 | None |
| PF linear trend | None |
| PF deviation (Hz) | Log |

Table S1. Transformations applied to the raw data of acoustic parameters prior to z-transformation and LDA

To check the multivariate assumptions of LDA we ran a MANOVA, with population as the independent variable and the Z-scores of spectral parameters as dependant variables. We visually inspected plots of residuals to assess homogeneity and normality, and plotted mahalanobis distance to assess multivariate normality; data indicated no obvious deviations from these assumptions.

**S2. Individual differences in call structure**

Before running LDA we carried out the same tests as described above to ensure that the 29 parameters met univariate and multivariate assumptions. Table S2 gives details on transformations applied prior to analyses.

| **Parameter** | **Transformation** |
| --- | --- |
| Ex1 duration | Log |
| F0 | None |
| F0start | None |
| F0end | None |
| F0 linear trend | +3 then square root |
| Tonality | +3 then log |
| First quartile | None |
| First quartile_1 | ^ 2 |
| First quartile _2 | None |
| First quartile _3 | None |
| First quartile _4 | None |
| Second quartile | ^ -1 |
| Second quartile _1 | None |
| Second quartile _2 | ^ -0.8 |
| Second quartile _3 | None |
| Second quartile _4 | None |
| Third quartile | None |
| Third quartile_1 | None |
| Third quartile _2 | None |
| Third quartile _3 | None |
| Third quartile _4 | None |
| Frequency range | None |
| Peak frequency | ^ 1.9 |
| Peak frequency_1 | ^ -0.6 |
| Peak frequency_2 | None |
| Peak frequency_3 | None |
| Peak frequency_4 | None |
| PFlinear trend | +8 then ^ 1.8 |
| PFdeviation (Hz) | +3 then log |

Table S2. Transformations applied to the raw data of acoustic parameters prior to z-transformation and LDA

**S3. Behavoural responses to bark vocalisations**

We visually inspected boxplots and histograms of non-binomial behavioural responses to assess heterogeneity of variance between conditions, approximate symmetric distribution and absence of outliers. Following this we fitted models (minus random effects) using the function glm (applying the transformations and GLMM family described in table S3a), and determined variance inflation factors (VIFs) using the vif function of the R package car (Fox and Weisberg 2011), and visually assessed graphs illustrating the distribution of model residuals. VIFs indicated that collinearity was not an issue (largest VIF = 1.05). Variables run with gaussian error structure met the assumptions of normally distributed and homogenous residuals. Maximum distance travelled by the subject within 30 seconds did not meet these assumptions and we ran this model using a poisson error structure. Original data were overdispersed so we transformed these measures to ordinal data. Following this, data was still somewhat overdispersed (dispersion parameter = 2.1), but as overdispersion can lead to tests becoming anti-conservative, results indicating a non-significant difference (as we found in this case) are still reliable.

Table S3a. Transformations applied to the raw data of behavioural measures prior to statistical analysis, and family of GLMM applied

| GLMM | Transformation | GLMM family |
| --- | --- | --- |
| Duration of first look towards speaker | Square root | Gaussian |
| Maximum distance within 30 seconds | Ordinal | Poisson |
| Subject's height within 30 seconds | None | Binomial |
| Subject's height over 30 minutes | None | Binomial |
| Proximity to loud speaker after 3 minutes | None | Gaussian |
| Minimum distance to loud speaker over 30 minutes | None | Gaussian |
